# From Waste to Health: Olive Mill Wastewater for Cardiovascular Disease Prevention

**DOI:** 10.3390/nu16172986

**Published:** 2024-09-04

**Authors:** Laura Beatrice Mattioli, Ivan Corazza, Roberta Budriesi, Silvana Hrelia, Marco Malaguti, Cristiana Caliceti, Rosa Amoroso, Cristina Maccallini, Pasquale Crupi, Maria Lisa Clodoveo, Marilena Muraglia, Alessia Carocci, Roberta Tardugno, Alexia Barbarossa, Filomena Corbo

**Affiliations:** 1 Food Chemistry and Nutraceutical Lab, Department of Pharmacy and Biotechnology, Alma Mater Studiorum, University of Bologna, 40126 Bologna, Italy; laura.mattioli13@unibo.it; 2 Department of Medical and Surgical Science (DIMEC), Alma Mater Studiorum, University of Bologna, 40138 Bologna, Italy; ivan.corazza@unibo.it; 3 Department for Life Quality Studies, Alma Mater Studiorum, University of Bologna, Corso d’Augusto 237, 47921 Rimini, Italy; silvana.hrelia@unibo.it (S.H.); marco.malaguti@unibo.it (M.M.); 4 Department of Biomedical and Neuromotor Sciences, Alma Mater Studiorum, University of Bologna, 40126 Bologna, Italy; cristiana.caliceti@unibo.it; 5 Department of Pharmacy, University ‘G. d’Annunzio’ of Chieti-Pescara, 66100 Chieti, Italy; rosa.amoroso@unich.it (R.A.); cristina.maccallini@unich.it (C.M.); 6 Department of Agricultural, Food and Forestry Sciences, University of Palermo, V. Le Delle Scienze 13, 90128 Palermo, Italy; pasquale.crupi@unipa.it; 7 Interdisciplinary Department of Medicine, School of Medicine, University of Bari ‘Aldo Moro, 70124 Bari, Italy; marialisa.clodoveo@uniba.it; 8 Department of Pharmacy—Drug Science, University of Bari ‘Aldo Moro’, 70125 Bari, Italy; marilena.muraglia@uniba.it (M.M.); alessia.carocci@uniba.it (A.C.); roberta.tardugno@uniba.it (R.T.); alexia.barbarossa@uniba.it (A.B.); filomena.corbo@uniba.it (F.C.)

**Keywords:** Momast Plus 30 Bio, *Olea europaea* L. cultivar *Coratina*, endothelial cells, antioxidant effects, iNOS inhibition, inotropy chronotropic and vasorelaxant activities, arterial stiffness, vascular aging, antimicrobial activity

## Abstract

Waste from the agri-food chain represents a valuable reservoir of organic compounds with health-promoting properties. **Momast Plus 30 Bio (MP30B)** is a derivative obtained from olive-oil wastewater. Its enrichment in hydroxytyrosol (HT) via a patented technique has paved the way for its potential application as a dietary supplement in preventing cardiovascular diseases. **MP30B** demonstrates no significant alteration in cardiac and vascular parameters in “ex vivo” studies. However, it exhibits a strong ability to remove reactive oxygen species and exerts anti-inflammatory effects, notably reducing the concentration of iNOS and mitigating heart infections in “in vitro” experiments. Furthermore, **MP30B** slightly decreases the stiffness of the “ex vivo” thoracic aorta, potentially resulting in lowered arterial pressure and enhanced energy transfer to a normal ventricle. Based on these findings, we posit **MP30B** as a promising extract for cardiovascular disease prevention, and its specific antibacterial properties suggest its utility in preventing cardiac infections.

## 1. Introduction

Waste products from the agri-food agricultural and food-producing chain have long been considered a source of waste that is difficult to dispose of [1]. Despite everything, this type of waste represents an exciting resource: these biomasses are rich in organic compounds with multiple uses. The accumulation of biomass has a substantial impact on the climate and human health [2]; therefore, adopting a circular economy approach might bring enormous advantages in both the economic and health fields [3]; indeed, in the last decades numerous studies and projects have made it possible to exploit these wastes in a circular economy perspective, as food for zootechnical purposes, as a source of biofuel, but also as a supplier of bioactive molecules. In the nutraceutical field, in fact, for instance, it is possible to exploit by-products from the agri-food chain as a matrix to extract mixtures of secondary metabolites for applications in the prevention and integrated medicine, food, supplement, or cosmetic production [4,5,6]. In this scenario, waste or by-products from many plant processes are demonstrated to contain health-promoting substances to be exploited as antioxidant, chemopreventive, cardioprotective, or even neuroprotective agents [6,7,8].

Therefore, studies in the nutraceutical field are of particular importance, as they allow the characterization of the bioactive potential of these extracts and contribute to the valorization of waste as a resource from a circular economy perspective [9].

Our research group has been working for a long time in the field of characterization and valorization of extracts obtained from waste for applications in the cardiovascular [10], gastrointestinal [11], antioxidant [12,13,14], and neuroprotective fields [6,15].

The secondary products of olive oil mill preparation (as leaves or wastewater) have long been studied as a supplier of molecules with anti-inflammatory and antioxidant action [16]. Olive leaf extracts are, for example, used in pharmaceutical preparations for hypotensive and hypoglycemic purposes [17,18]. Moreover, an extract obtained from olive leaves, characterized by high secoiridoids content, has shown interesting activities related to the cardiovascular system [19], allowing the development of products with a protective action on the latter [20].

Olive oil extraction leads to considerable waste, including olive mill wastewater (OMWW), which is estimated to reach 30 million cubic meters annually in the Mediterranean area. OMWW poses an important waste management problem since the lack of conventional treatment can counteract its high toxicity. However, phenolic compounds in the OMWW represent an opportunity to “upcycle” this waste to obtain high-added-value products [21].

Recently, our research group studied extracts obtained from OMWW from the *Coratina* cultivar. Results highlighted potential application in Inflammatory Bowel Syndrome (IBS) as these extracts modulate various targets related to the disease. In addition, the effects are linked to the map of the secondary metabolites present, which is linked in turn to the waste treatment methods [22].

**Scheme 1:**
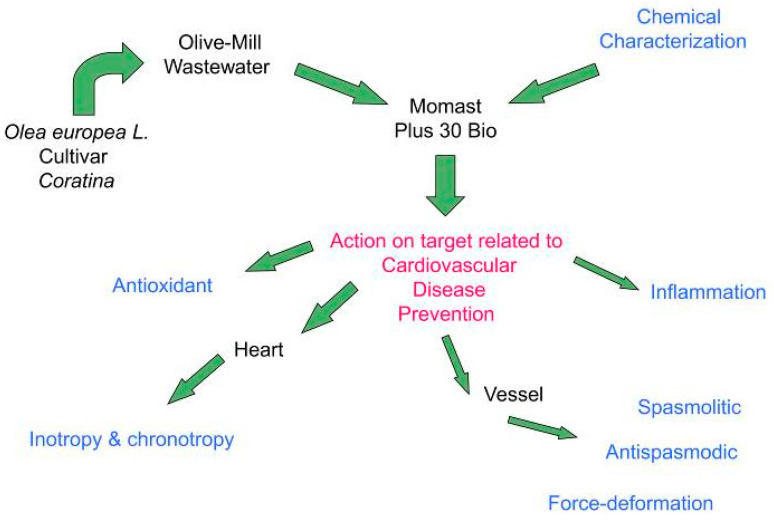
Research graphical summary.

Combining these indications and considering that the network of cardiovascular targets intersects with that of gastrointestinal diseases, we focused our attention on the possibility of exploiting an extract from cv. *Coratina* OMWW, namely, MOMAST Plus 30 Bio (MP30B), to be used for cardiovascular protection (Scheme 1). In detail, in the present work, the action of the **MP30B** has been studied on cardiac activity, evaluating its effect on atrial contractility (inotropic and chronotropic effects) and vascular and antispasmodic activity. The antioxidant action of the polyphenols is well-known. Given the high concentration of polyphenols in the **MP30B**, an evaluation of the antioxidant and anti-inflammatory action on the vascular endothelial cells was conducted, together with the investigation of the inducible Nitric Oxide Synthase (iNOS) activity.

As a corollary to evaluating its effects on the heart and vascular endothelium, considering the previously highlighted antimicrobial action against strains found in chronic intestinal pathologies such as IBS, as demonstrated for **MP30B** [22], its potential efficacy was also investigated against bacterial strains (especially streptococci) responsible for myocarditis. Due to the growing microbial resistance, these strains are increasingly less responsive to common antibiotics.

## 2. Materials and Methods

### 2.1. Wastewater Plant Material

**MP30B** is a polyphenolic liquid complex derived from wastewater olive oil’s mechanical filtration process. It represents a fraction of MOMAST^®^, a patented natural phenolic complex [23] enriched in tyrosol and hydroxytyrosol (HT). **MP30B** is obtained by a mechanical process based on micro-, nano-, and ultrafiltration from the Apulian olive (*Olea europaea* L. cv. *Coratina*). It was provided by Bioenutra SRL (Ginosa, Taranto, Italy).

### 2.2. Chemical Analysis

Chemical analysis of organic and inorganic residues in the extract **MP30B** was carried out by an accredited laboratory (ACCREDIA LAB n. 0648) in the Basilicata region (Marconia, Italy) following UNI CEI ISO/IEC 17025:2018 regulations. An HPLC 1200 (Agilent Technologies, Palo Alto, CA, USA) equipped with a degasser, quaternary pump solvent delivery, thermo-stated column compartment, and a diode array detector (DAD) was employed to determine the phenolic compounds profile of **MP30B**. Chromatographic separation was performed as reported in [22]. Phenolics were identified by matching their absorption characteristics and retention times with those from analytical standards (gallic acid, 3-hydroxytyrosol, tyrosol, verbascoside, and oleuropein provided by Sigma Aldrich, Milan, Italy). Calibration curves in the concentration range of 1–1000 µg/mL, depending on the content of the different phenolics in the real sample, were performed through the internal standard (syringic acid) method and used to quantify the identified compounds. The results of triplicates (means ± standard deviation) were expressed as mg/g of extract (**MP30B**).

### 2.3. Determination of the Total Phenolic Content (TPC)

**MP30B** total phenolic content (TPC) was determined by adopting the Folin–Ciocalteu method previously described [13,14]. In particular, the Folin–Ciocalteu (F-C) reduction ability was evaluated using a microplate reader (Tecan Infinite Pro 200 Microplate Reader, Tecan Group Ltd., Mannedorf, Switzerland) with spectrophotometric detection and 96-well microtiter plates (Greiner 96 Flat Bottom Transparent Polystyrene). Therefore, 12.5 µL of a standard gallic acid (GA) solution or diluted MP30B was added to each well. Subsequently, 12.5 µL of methanol (MeOH) in 50 µL of distilled water, 12.5 µL of the Folin–Ciocalteu reagent, and 112.5 µL of distilled water were added to each well. Following a five-minute incubation period, 50 µL of a 20% Na_2_CO_3_ solution was added to each well, and the microplate was incubated for a further 90 min at 30 °C. The absorbance values were then registered at λ = 700 nm. A series of gallic acid (GA) standard solutions at different concentrations (0.025–0.25 mg/mL) was prepared and used to construct a calibration curve (y = 3.3278x − 0.0045; R^2^ = 0.9974). To account for any potential interference from the samples and standard, methanol was used as a blank. The total phenolic content (TPC) value was expressed as milligrams of gallic acid equivalent (GAE) per gram of **MP30B** (mg GAE/g of MP30B).

### 2.4. Antioxidant Assay: DPPH Assay Procedure

The free radical scavenging ability of **MP30B** was assessed through the 2,2-diphenyl-1-picrylhydrazyl (DPPH) radical scavenging assay previously detailed in the literature [13,14]. The **MP30B** antioxidant profile was analyzed using a microplate reader (Tecan Infinite Pro 200 Microplate Reader, Tecan Group Ltd., Mannedorf, Switzerland with spectrophotometric detection and 96-well microtiter plates (Greiner 96 Flat Bottom Transparent Polystyrene).

In each well, 162.5 µL of DPPH solution (0.1 mM in MeOH) freshly prepared was added to 87.5 µL of samples in MeOH at varying concentrations (0.5–0.005 mg/mL). The reaction mixture was allowed to incubate at 30 °C for 30 min. A control sample was prepared by mixing 87.5 µL of methanol with 162.5 µL of a DPPH solution. The absorbance of the mixture was estimated spectrophotometrically at a wavelength of 517 nm.

The following equation was employed to estimate the percentage of DPPH radical scavenging activity:

% DPPH radical scavenging activity = (A_control_ − A_sample_)/A_control_ × 100

where “A_control_” is the absorbance of the control and “A_sample_” is the **MP30B** absorbance.

Antiradical curves were generated, with concentration on the x-axis and % DPPH radical scavenging activity on the y-axis. SC_50_ (µg/mL necessary for a 50% reduction of the DPPH radical) was then calculated from the graph. The experiment was repeated three times at each concentration.

### 2.5. Ex Vivo Cardiovascular Experiments

#### 2.5.1. Animals

Guinea pigs (200–400 g) obtained from Charles River (Calco, Como, Italy) were used. The animals were housed according to the ECC Council Directive regarding protecting animals for experimental and other scientific purposes (Directive 2010/63/EU of the European Parliament and of the Council) and the WMA Statement on Animal Use in Biomedical Research. The work was conducted according to the guidelines set forth by EU Directive 2010/63/EU and ARRIVE guidelines [24,25]. All procedures followed the guidelines of the animal care and use committee of the University of Bologna (Bologna, Italy). The ethical committee authorization was reported and numbered as “Protocol PR 21.79.14” by the Scientific Ethics Committee for Animal Research Protocols, according to D.L. vo 116/92. Guinea pigs were sacrificed by cervical dislocation. The organs studied were the heart (left and right atria) and aorta. The organs were set up rapidly and used as described below.

#### 2.5.2. Guinea Pig Heart

After thoracotomy, the heart was immediately removed and washed by perfusion through the aorta with oxygenated Tyrode solution containing (mM): NaCl 136.9; KCl 5.4; CaCl_2_ 2.5; MgCl_2_ 1.0; NaH_2_PO_4_xH_2_O 0.4; NaHCO_3_ 11.9; and glucose 5.5. Spontaneously beating right atria and left atria driven at 1 Hz were used. The left and the right atria were dissected from the ventricles, cleaned of excess tissue, and hung vertically in a 15 mL organ bath containing the physiological salt solution (PSS) continuously bubbled with 95% O_2_—5% CO_2_ at 35 °C, pH 7.4. The contractile activity was recorded isometrically using a force transducer (FT 0.3, Grass Instruments Corporation, Quincy, MA, USA) using Power Lab^®^ software (AD-Instruments Pty Ltd., Castle Hill, Australia, Lab, Chart 7 Pro). The left atria were stimulated by rectangular pulses of 0.6–0.8 ms duration and about 50% threshold voltage through two platinum contact electrodes in the lower holding clamp (Grass S88 Stimulator). The right atria were in spontaneous activity.

Inotropic and chronotropic activities. After several min, on the left and right atria prepared as described above, a length–tension curve was determined, and the muscle length was maintained at the value that elicited 90% of the maximum contractile force observed at the optimal length. After a 45–60 min stabilization period, the atria were challenged by various agents. During the equilibration period, the bathing solution was changed every 15 min, and the threshold voltage was ascertained for the left atria. Atrial muscle preparations were used to examine the inotropic and chronotropic activity of **MP30B** (0.01–10 mg/mL), water dissolved. The increasing extract concentrations were added after a steady state was reached during the generation of cumulative concentration–response curves. One set of experiments was carried out using nifedipine (0.01–1 µM) as positive control.

#### 2.5.3. Guinea Pig Aorta

The thoracic aorta was removed and placed in Tyrode solution containing (mM): NaCl, 118; KCl 4.75; CaCl_2_ 2.54; MgSO_4_ 1.20; KH_2_PO_4_ 1.19; NaHCO_3_ 25; and glucose 11; and bubbled with 95% O_2_—5% CO_2_, pH 7.4. The vessel was cleaned of extraneous connective tissue. Two helicoidal strips (10 mm × 1 mm) were cut from the aorta beginning from the end proximal to the heart (thoracic and abdominal portions). Vascular strips were then tied with surgical thread (6-0) and suspended in 15 mL of aerated PSS at 35 °C in a jacketed tissue bath. Aortic strips were secured at one end to plexiglass hooks and connected via the surgical thread to a force-displacement transducer (FT 0.3, Grass Instruments Corporation, Quincy, MA, USA) for monitoring changes in isometric contraction and washed every 20 min with fresh PSS for one h.

Spasmolytic activity against K^+^ (80 mM) depolarization. As previously described [19], thoracic aortic strips were subjected to a resting force of 1 g. After recovery, guinea pig aortic strips were contracted by washing in PSS containing 80 mM KCl (equimolar substitution of K^+^). When the contraction reached a plateau (about 45 min or 15 min, respectively), different concentrations of the MP30B (0.01–10 mg/mL) were added cumulatively to the bath, allowing for any relaxation to obtain an equilibrated level of force. In the same experimental condition, cumulative concentration curves to Nifedipine (0.001–1 µM), used as a positive control, were performed.

Antispasmodic activity vs. K^+^. Strips from the thoracic aorta were separately used. After stabilization for one hour, with changes in the bathing solution, every 15 min, cumulative concentration–response curves were obtained by adding potassium chloride (K^+^) (20–80 mM). Following incubation with the **MP30B** (1 mg/mL) or in the presence of Nifedipine (5.0 nM) for 30 min, a new concentration–response curve to K^+^ was obtained. Parallel experiments in the absence of **MP30B** or nifedipine used as the antagonist were run.

Effect on aorta spontaneous contraction. The graphs of spontaneous thoracic aorta smooth muscle contractions were recorded with the LabChart 7.0 Pro Software (AD Instruments, Bella Vista, New South Wales, Australia) using the previously described method [11]. After a period of 30 to 45 min according to each tissue, cumulative concentration curves of **MP30B** were constructed. A 5-minute stationary interval of the Spontaneous Contraction (SC) recording was selected at the final stage of each concentration. For each interval, the following parameters were extracted and calculated:

- The Mean Contraction Amplitude (MCA), evaluated as the mean force value (g);
- The standard deviations of the force values over the period as an index of the Spontaneous Contraction Variability (SCV);
- The Basal Spontaneous Motor Activity (BSMA) as the percentage (%) variation in each mean force value (g) for the control period.

The spontaneous contractions were investigated in the frequency domain through a standard FFT analysis and a subsequent Power Spectral Density (PSD) plot. The absolute powers of the following frequency bands of interest—low [0.0,0.2 [Hz (LF), medium [0.2,0.6 [Hz (MF), and high [0.6,1.0] Hz (HF) [11]—were then calculated. The PSD percentage (%) variations for each band of interest for control were estimated.

All of the calculations were carried out in a post-processing phase. A skilled operator chose the analysis period to avoid errors due to artifacts.

Force–Deformation curves. Three tissue samples were prepared, three from the thoracic aorta and three from the thoracic and abdominal aorta. Samples were taken by cutting the aorta in a spiral and uniformly sized (length 6 mm; width 4 mm). The thickness was considered uniform and constant for all samples. Each sample was then studied relative to its length to evaluate its elastance along the circumference of the aorta (to analyze the ability of the aorta to change its volume).

The samples were stored in a Tyrode solution with the composition detailed above. One sample for each aortic tract was used as control, while the other two were each immersed in solutions with **MP30B** at 1 mg/mL and Nifedipine at 5.0 nM as positive control.

Each sample was then inserted into an electromechanical device [26] equipped with a strain gauge (Transducer C2G1-B) capable of applying traction forces and stretching the tissue in a controlled manner. The forces exerted, and the elongations were acquired with the Anscovery System (SparkBio Srl, Bologna, Italy). The elongation of the sample was referred to as the initial length, and the deformation was thus obtained (ε = ΔL/L_0_).

Since there is a quadratic relationship between the circumference length and its area (proportional to the volume), the corresponding volume quadruples if the circumference doubles (a condition in which ε = 1).

Traction was applied until specimen failure. Then, the force–strain curves were constructed.

### 2.6. Effect on “In Vitro” Vascular Endothelial Function

#### 2.6.1. Cell Culture

The e effect of **MP30B** on endothelial function was meticulously studied through a series of experiments conducted in human umbilical vein endothelial cells (HUVECs), a well-known in vitro model for studying endothelial cell physiology [27]. The commercially available HUVEC pools (Life Technologies, Thermo Fisher, Waltham, MA, USA) were carefully seeded on gelatin-coated tissue culture dishes and maintained in phenol red-free basal medium M200 (Life Technologies) supplemented with 10% Fetal Bovine Serum (FBS) and growth factors (LSGS, Life Technologies) at 37 °C with 5% CO_2_. The cells from passages 3 to 7 were actively increasing (70–90% confluent) when samples were harvested and analyzed [28].

#### 2.6.2. Cell Viability Bioassay

The cell viability was performed by using WST8 [2-(2-methoxy-4-nitrophenyl)-3-(4-nitrophenyl)-5-(2,4-disulfophenyl)-2H-tetrazolium, monosodium salt] (Dojindo Molecular Technologies, Japan). In the presence of an electron mediator, WST8 is reduced to the red formazan dye by intracellular dehydrogenases, which are soluble in the cell medium, thus directly proportional to living cells [29]. HUVECs were seeded in a transparent 96-well plate at 104 cells/well density. To assess the cell viability, a stock solution of **MP30B** was prepared by 1:50 dilution in PBS, and the subsequent dilutions for cell treatments were made in the cell medium. HUVECs were treated for 24 h with different concentrations of **MP30B** (range 260–0.26 μg GAE/mL). The decrease in absorbance between 24 h treatment and the control was assessed at 37 °C at 450 nm using a Varioskan™ flash multimode reader (Thermo Fisher, Waltham, MA, USA).

#### 2.6.3. Intracellular Total Oxidant Fluorescent Detection

Intracellular oxidant levels were investigated using the oxidant-sensitive fluorescent probe 2′,7′-dichlorodihydrofluorescein diacetate (H_2_DCFDA). Indeed, the fluorescent response based on the oxidation of DCFH caused, for example, by changes in iron or heme signaling or peroxynitrite (ONOO-) formation provides an index for the total oxidants present in biological systems, not just for cell-derived H_2_O_2_ [30,31].

Briefly, the probe is not fluorescent until intracellular esterases remove the acetate groups; in the presence of oxidants, the probe is oxidized within the cells, producing a fluorescent signal related to intracellular oxidant levels that were measured using a microtiter plate reader (Varioskan™ flash multimode reader, Thermo Fisher Scientific, Waltham, MA, USA). The excitation and emission wavelengths were 485 nm and 535 nm, respectively. HUVECs were treated with **MP30B** (26–0.26 μg GAE mg/mL) for 24 h, and 100 μM HT was used as a positive control. After 24 h treatment, cells were exposed to 5 μM H_2_DCFDA for 20 min at 37 °C and then injured by 50 μM H_2_O_2_ for 30 min. The decrease in fluorescence level (AU) between cells pretreated with **MP30B** and untreated was reported as the IC_50_ of intracellular reactive species (RS) normalized with the TPC in μg/GAE/mL [32].

#### 2.6.4. RNA Extraction

HUVECs (1.5 × 10^5^ cells/well) were seeded in six-well plates and incubated with **MP30B** (2.6 μg GAE/mL) for 8 h at 37 °C before 24 h of TNF-α (10 ng/mL) treatment. Total RNA was extracted using a commercially available kit (QIAGEN) [33]. RNA concentration and purity were determined by NanoDrop 2000 spectrophotometer (Thermo Fisher Scientific, Waltham, MA, USA).

#### 2.6.5. Real-Time PCR

Briefly, 25 ng of total RNA was reverse transcribed using the SuperScript^®^ III First-Strand Synthesis SuperMix (Life Technologies, Carlsbad, CA, USA) and then amplified using the EXPRESS SYBR^®^ GreenER™ qPCR SuperMix (Life Technologies, Carlsbad, CA, USA) according to the manufacturer’s protocol in a final volume of 20 μL. Real-time PCR reactions were performed on a Rotor-Gene Q QIAGEN Real-Time PCR System (QIAGEN GmbH, QIAGEN Strasse 1, D-40724, Hilden), the protocol includes an initial 5 min incubation at 60 °C, then 2 min at 95 °C, followed by 40 cycles of amplification: 95 °C for 15 s and 60 °C for 1 min and examined on by Rotor-Gene Real-Time Analysis Software 6.0 (QIAGEN GmbH, QIAGEN Strasse 1, D-40724, Hilden) [34]. The primer concentration was 500nM. The following primers were used: VCAM: forward 5′-GGTATCTGCATCGGGCCTC-3′, reverse 5′-TAAAAGCTTGAGAAGCTGCAAACA-3′; and RPL13A: forward 5′-CACCCTGGAGGAGAAGAGGA-3′, reverse 5′-CCGTAGCCTCATGAGCTGTT-3′. Changes in gene expression were calculated by the 2^−ΔΔCt^ formula using RPL13A as a reference gene.

### 2.7. Determination of iNOS Inhibition in a Cell-Free System

#### 2.7.1. General

To evaluate iNOS, a Waters (Milford, MA, USA) HPLC system composed of a P600 model pump, a 2996 photodiode array detector, and a 7725i model sample injector (Rheodyne, Cotati, CA, USA) and a column thermostat oven module Igloo-Cil (Cil Cluzeau Info Labo, France) was used. The Empower Pro v.2 software (Waters) was used to process data. The analyses were performed on an XTerra MS C8 column (250 × 4.6 mm id, 5 μm particle size) (Waters), equipped with an XTerra MS C8 guard column (Waters). Linear gradient of buffers A (5% CH_3_CN in 15 mM sodium borate with 0.1% *v*/*v* trifluoroacetic acid, pH 9.4) and B (50% CH_3_CN in 8 mM sodium borate with 0.1% *v*/*v* trifluoroacetic acid, pH 9.4) were used as the mobile phases, and the flow rate was 0.7 mL/min. The solvent gradient was 0–20% B at 0–10 min, B to 25% at 10–15 min, 40% at 15–20 min, and 70% at 20–35 min. The injection volume was 5 μL. The fluorescence detection of the column eluate was performed at 335 nm (excitation) and 439 nm (emission).

#### 2.7.2. Procedure

Recombinant murine iNOS was purchased from Cayman Chemical (Ann Arbor, MI, USA). The enzyme stock solution (10 μL) was added to 80 μL of 2-[4-(2-hydroxyethyl)piperazin-1-yl]ethanesulfonic acid (HEPES) buffer pH = 7.4, 100 mM, containing 0.1 mM CaCl_2_, 1 mM D,L-dithiothreitol (DTT), 0.5 mg/mL BSA, 10 μM flavin mononucleotide (FMN), 10 μM flavin adenine dinucleotide (FAD), 30 μM tetrahydrobiopterin (BH_4_), 10 μg/mL calmodulin (CaM), 10 μM L-Arg. Then, 10 μL of the **MP30B** solution (100–1 mg/mL) or the positive control 1400W (1 mM) was added to the assay mixture. After a pre-incubation time of 15 min at 37 °C, each reaction was initiated by the addition of 10 μL of nicotinamide adenine dinucleotide phosphate (NADPH) 7.5 mM, and it was carried out at 37 °C for 30 min. Reactions were then stopped by adding 500 μL of ice-cold CH_3_CN. Before being analyzed by HPLC, the solutions were dried under a vacuum and subjected to fluorescence derivatization [35]. The content of the L-citrulline (L-Cit) was evaluated for the Nitric Oxide Synthase (NOS) reaction control, where no inhibitor was added (0% inhibition), whereas 1400 W was the reference compound (100% inhibition).

### 2.8. Antimicrobial Studies

#### 2.8.1. Microorganisms

The antibacterial activity was tested against six streptococcal strains, of which five belonged to the American Type Culture Collection (ATCC, Rockville, MD, USA), and one was derived from clinical isolation. Strains from the ATCC were *Streptococcus mutans* (ATCC 25175), *Streptococcus pneumoniae* (ATCC 10015), *Streptococcus pyogenes* (ATCC 19615), *Streptococcus salivarius* (ATCC 13419), and *Streptococcus sanguinis* (ATCC 10556). The clinical isolate was from patients admitted to the Department of Biomedical Science and Human Oncology intensive care unit, University of Bari, Italy. The isolation and identification procedures were conducted in the Hygiene Section of the Department. Using conventional physiological and morphological methods (API systems), the strain was identified as *S. pyogenes FL*. Blood Agar Base (BAB) supplemented with 5% defibrinated horse blood and Brain heart infusion broth (BHI) was used for growing bacteria throughout the study. Müller–Hinton (MH) agar and cation-adjusted MH (CAMH) broth were used in susceptibility testing, supplemented with 5% lysed horse blood (Oxoid, Italy). All strains were grown and maintained at 37 °C.

#### 2.8.2. Determination of MIC and MBC

The Minimum Inhibitory Concentration (MIC) was determined by the broth microdilution method as recommended by CLSI [36]. A bacterial sample was taken from previously cultured Petri dishes and diluted in test tubes containing 3 mL of CAMH broth supplemented with 5% lysed horse blood. Then, serial dilutions were performed with 100 µL of the test solutions to obtain concentrations ranging from 130 to 0.1 mg/mL for **MP30B**, prepared using 1 mL stock solution added to 4 mL MHB, to obtain the concentration solution 260 mg/mL, until the penultimate well and the last one were able to control microbial growth [37]. The plates were then incubated at 37 °C for 24 h. The MIC was defined as the lowest concentration at which no microbial growth was observed. MBCs were determined by subculturing, onto horse blood agar plates, 10 μL of broth from wells containing antimicrobial concentrations greater than or equal to the MIC of the corresponding compound. Plates were incubated at 37 °C for 48 h, and viable colonies were counted. The MBC was defined as the lowest concentration of drug-yielding colony counts <0.1% of the initial inoculum (determined by colony counts from the growth control well immediately after inoculation). MICs and MBCs determinations were realized in triplicate in three independent assays.

### 2.9. Statistical Analysis

All data from cardiac parameters activity are reported as means ± SEM. The potency expressed as EC_50_ or IC_50_ values was calculated from concentration–response curves [38].

For the spasmolytic activity on K^+^-depolarized aortic strips, data were analyzed using Student’s *t*-test and are presented as mean ± S.E.M. [38]. Since the drugs were added cumulatively, the difference between the control and the experimental values at each concentration was tested for a *p* value < 0.05. The potency of drugs defined as EC_50_ or IC_50_ was evaluated from concentration–response curve (**MP30B**) or log concentration–response curves (Nifedipine/Potassium chloride) [Probit analysis using Litchfield and Wilcoxon [38]] and GraphPad Prism^®^ 5.0 software [39,40] in the appropriate pharmacological preparations.

For changes in spontaneous contractility, all the calculations were performed in a post-processing phase with the Lab Chart 7 pro Software. A skilled operator chose the analysis period to avoid errors due to artifacts. Comparisons between mean spontaneous contraction amplitudes (MCA) at different concentrations were performed by one-way ANOVA with Bonferroni’s correction. Differences with *p* < 0.05 were considered statistically significant. LF, MF, and HF contractions were deemed significant when variations were higher or lower than 50% for the basal values.

Graphical analysis was performed using the GraphPad Prism 5.0 (GraphPad Software Inc., San Diego, CA, USA) for the antioxidants results.

Results for the effect on vascular endothelial function are expressed as mean ± SD of at least three independent experiments. Differences between the means were determined by unpaired student’s *t*-test or one-way ANOVA followed by Bonferroni multiple comparison test using the GraphPad Prism Software, version 6.0 (GraphPad Software, Inc., La Jolla, CA, USA).

## 3. Results

### 3.1. Chemical Assay for the Measurement of the Total Polyphenolic Content (TPC) and Scavenging Activity of MP30B

Since we want to test the effect of **MP30B** on cardiovascular protection and propose it as a nutraceutical extract, a series of chemical analyses to determine the absence of pollutants is appropriate. Table S1 (Supplementary Materials) lists the content of inorganic and organic contaminants in **MP30B** quantified by accredited methods following UNI CEI ISO/IEC 17025:2018 regulations; it is worth noting that the analyzed compounds’ concentrations were mostly lower than their detection limits. Therefore, the extract did not present any toxic compounds, especially heavy metals, aromatic polycyclic hydrocarbons, and persistent organic pollutants.

Moreover, the main phenolics, known to have cardiovascular and antimicrobic activities [41,42], were identified and quantified by HPLC-VWD at 280 nm (Figure S1, Supplementary Materials). Three simple phenols (gallic acid, 3-hydroxytyrosol, and tyrosol) were found in higher concentrations than the secoridoids oleuropein and verbascoside in **MP30B**.

The antioxidant potential of phenolic compounds in olive mill wastewater from producing extra virgin olive oil (EVOO) is well-known. The total phenolic content (TPC) of **MP30B** was determined using the Folin–Ciocalteu assay by measuring the absorbance value of the sample at λ = 700 nm and using a calibration curve with GA standard solutions (y = 3.3278x − 0.0045, R^2^ = 0.9974). The total polyphenolic content, expressed as milligrams of gallic acid equivalent (GAE) per gram of **MP30B**, was found to be 132.96 ± 2.09 mg GAE/g of **MP30B**.

The radical scavenging activity of MP30B was assessed using a DPPH assay in a microplate format, setting the absorbance value at λ = 517 nm. The radical scavenging activity SC_50_) is expressed in µg/mL necessary for a 50% reduction of 2,2-Diphenyl1-Picrylhydrazyl (DPPH) radical. Interestingly, gallic acid (3.1511 µg/mL), taken as a positive control, is only about four times more potent than **MP30B** (14.2935 µg/mL) as radical scavenging.

### 3.2. Cardiovascular Studies

The cardiovascular profile of **MP30B** was studied on guinea pigs isolated left and right atria to evaluate their inotropic and chronotropic effects, respectively, to assess the percent decrease in developed tension on isolated left atrium driven at 1 Hz (negative inotropic activity) and the percent decrease in the atrial rate on spontaneously beating right atrium (negative chronotropic activity). Spontaneous activity and elastance, spasmolytic and antispasmodic activities, on aortic strips using K^+^, were derived. **MP30B** was checked at increasing concentrations using Nifedipine as a positive control.

#### 3.2.1. Cardiac Activity

The data relating to the cardiac effects of **MP30B** and the positive control nifedipine are collected in Table 1a. **MP30B** has an interesting harmful inotropic intrinsic activity on the left atrium driven at 1 Hz at 1 mg/mL. The inotropic effect is also observed in spontaneous activity with significant intrinsic activity on the right atrium. The difference observed could be due to the frequency: the right atrium is, in fact, in spontaneous activity, and this indirectly influences the force of contraction. Regarding potency, the two data are not significantly different from each other. On the contrary, for **MP30B**, a weak positive chronotropic effect was observed (10%). The positive reference compound nifedipine has significantly adverse inotropic and chronotropic effects and higher potency.

#### 3.2.2. Vascular Activity

**MP30B** has been studied for its effects on vascular smooth muscles, its antispasmodic and spasmolytic activity against potassium-induced contraction, and its direct action on the tissue in spontaneous activity and elastance.

*Spasmolytic activity on guinea pig aorta smooth muscle (K^+^ 80 mM dep).* **MP30B** was studied for its spasmolytic activity on contraction induced by potassium 80 mM, using nifedipine as a positive control. **MP30B** has no relevant effects up to the maximum concentration studied. In contrast, nifedipine exhibits significant intrinsic activity (b in Table 1).

#### 3.2.3. Antispastic Activity on Guinea Pig Aorta Smooth Muscle vs. K^+^

**Figure 1:**
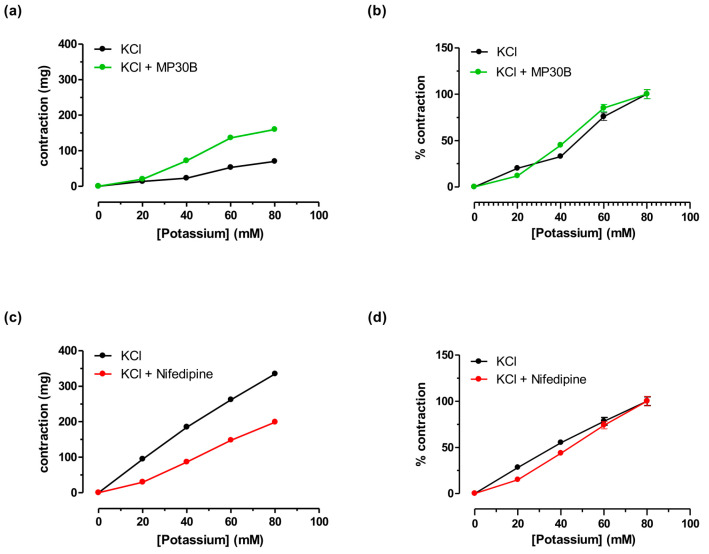
Cumulative concentration–response curves for potassium chloride before (black line) and after exposure of **MP30B** (1 mg/mL) (**a**,**b**) (green line) or nifedipine (0.005 µM) (**c**,**d**) (red line) for 30 min. on Guinea pig aortic strips. Data expressed as mg of developed contraction (**a**,**c**) and expressed as % of contraction (**b**,**d**). Each point is the mean ± SEM of three to five experiments. Where error bars are not shown, these are covered by the point itself.

**MP30B** was studied for its antispasmodic effects against potassium-induced contraction on aorta strips using nifedipine as a positive control. c in Table 1 contains the data relating to the mg of contraction developed by potassium in the absence or presence of **MP30B** or nifedipine and the developed potency. **MP30B** (1 mg/mL) and nifedipine (0.005 µM) do not modify the potency of potassium, expressed as IC_50_. The potencies, calculated for potassium alone and in the presence of **MP30B** (1 mg/mL) or nifedipine (0.005 µM), are not significantly different (c in Table 1) (Figure 1b,d). On the contrary, **MP30B** at the concentration used (1 mg/mL) increases the potassium-induced contraction by approximately 90%. In contrast, nifedipine (0.005 µM concentration) reduces the mg of potassium-induced contraction by approximately 40% (Figure 1a,c).

**Figure 2:**
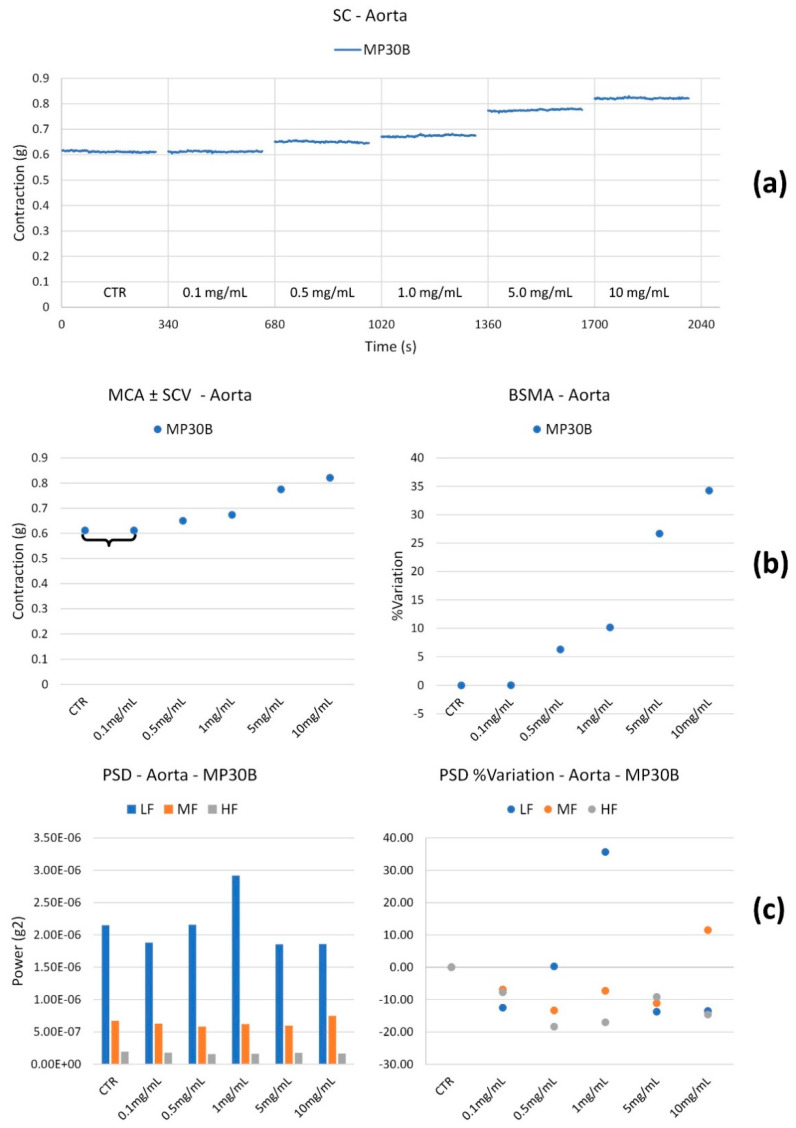
Experimental original recording of the concentration–response curve of **MP30B** on spontaneous thoracic aorta basal contractility: (**a**) Spontaneous contraction (**SC**) signals for each concentration; (**b**) mean contraction amplitude (**MCA**) and spontaneous contraction variability (**SCV**), represented as error bars in the **MCA** plot and contraction percentage variation for the control (**BSMA**) for each considered condition; not significant differences (*p* > 0.05) between **MCAs** at different concentrations are reported in the graph. All the comparisons not reported are to be considered significant (*p* < 0.05); (**c**) absolute powers (**PSD**) of the different bands of interest (**LF**: (0.0, 0.2) Hz; **MF**: (0.2, 0.6) Hz; **HF**: (0.6, 1.0) Hz) and PSD% variations for the control phase.

Effect on spontaneous activity on guinea pig aorta smooth muscle. On vascular smooth muscles in spontaneous activity, **MP30B** significantly modifies the tone for concentrations greater than 0.1 mg/mL, up to a maximum increase of 35% for concentrations of 10 mg/mL. Differences in frequency content (LF, MF, and HF) can be considered negligible since they are lower than the cut-off of 50% (Figure 2).

**Figure 3:**
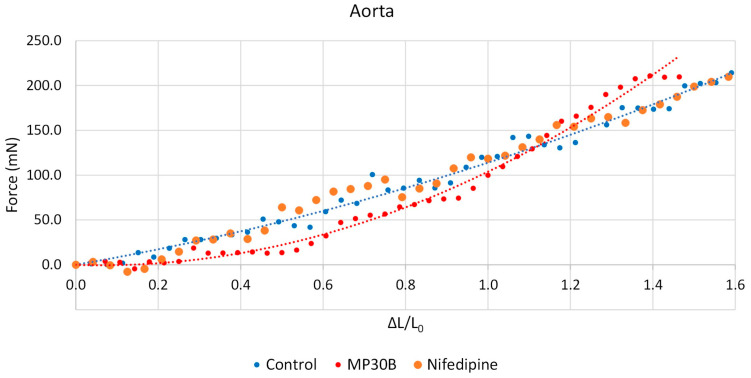
Force–deformation plots of the thoracic aorta samples in the different conditions (control and concentrations of 1 mg/mL for **MP30B** and 5.0 nM for nifedipine).

*Effect on force–deformation.* The graphs in Figure 3 show the force–deformation curves relating to each sample in the different conditions.

**MP30B** 1 mg/mL seems to produce a slight decrease in stiffness for low force values (<100 mN), probably leading to a reduction in arterial pressure [43].

### 3.3. Antioxidant and Anti-Inflammatory Activities on Human Umbilical Vein Endothelial Cells

Firstly, HUVECs were treated for 24 h with **MP30B** (range 260–0.26 μg GAE/mL) to investigate its potential cytotoxicity. The concentration range of 26–0.26 μg GAE/mL was investigated to evaluate the antioxidant activity of **MP30B**. These concentrations align with their real concentration in the bloodstream after ingesting the supplement [16,44]. Indeed, HT, verbascoside, and oleuropein undergo microbiota-induced processes in the gut, thus affecting their structures and activity [45]. Moreover, HT, the main metabolite of oleuropein, has a low post-ingestion availability [46], so we used low concentrations of **MP30B** better to predict the real scenario in the circulatory system.

Next, the fluorescent DCFH2DA-based cell bioassay was utilized to evaluate the intracellular antioxidant activity of **MP30B**. Briefly, HUVECs were treated with **MP30B** solutions for 24 h (range 26–0.26 μg GAE/mL); then, on the day of the experiment, the cell medium was changed, and cells were exposed to the oxidant agent H_2_O_2_ (50 µM). After 30 min of H_2_O_2_ injury, the fluorescence emission related to the different **MP30B** treatments (range 26–0.26 μg GAE/mL) was evaluated by a dose–response curve, obtaining an IC_50_ = 0.12 ± 0.01. HT was used as the positive control, and a sample without antioxidants was also measured as the blank control.

To better clarify the protective effect of **MP30B** for TNF-α-induced endothelial dysfunction, we evaluated the gene expression of inflammatory markers related to NF-κB signaling.

**Figure 4:**
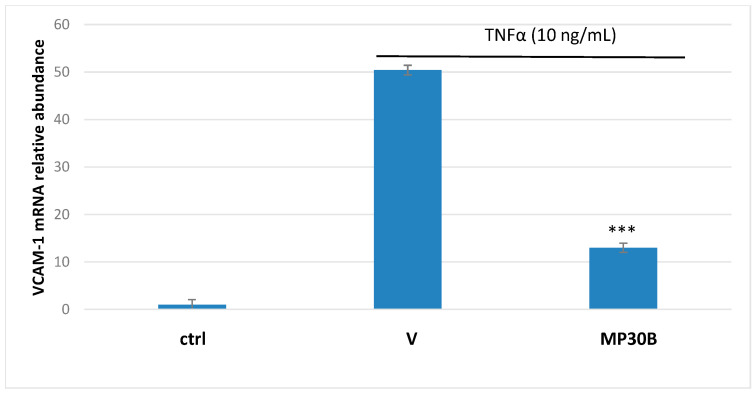
Effect of **MP30B** treatment on NF-kB signaling in HUVECs. HUVECs, starved overnight with phenol red-free M200 medium containing growth factors and 2% FBS, were pretreated with **MP30B** (2.6 μg GAE/mL) for 8 h before exposure to TNFα (10 ng/mL) for 24 h. Total RNA was extracted, and qRT-PCR analysis of VCAM and ICAM gene expression was performed. Relative changes in mRNA expression levels were calculated according to the 2^−ΔΔCt^ method using RPL13A as a reference gene. Results are expressed as mean ± SEM of five independent experiments, *** *p* < 0.001 significantly different from the vehicle (V, TNFα).

Classical NF-κB activity regulates the expression of many genes involved in inflammatory and survival responses, including those encoding cytokines (e.g., IL-1, IL-2, and IL-6), leukocyte adhesion molecules (e.g., E-selectin, ICAM-1, and VCAM-1), and antiapoptotic proteins (e.g., Bcl2, Bcl-XL, and XIAP) [29]. Therefore, the expression of the cellular adhesion proteins VCAM-1 and ICAM-1 was investigated. Briefly, HUVECs were treated with MP30B (2.6 μg GAE/mL) for 8 h and then injured with TNF-α for 24 h. As shown in Figure 4, **MP30B** pretreatment significantly decreased TNF-α-induced VCAM-1 expression (*p* < 0.001) in HUVECs while not affecting ICAM-1 expression.

### 3.4. iNOS Activity in a Cell-Free System

**Figure 5:**
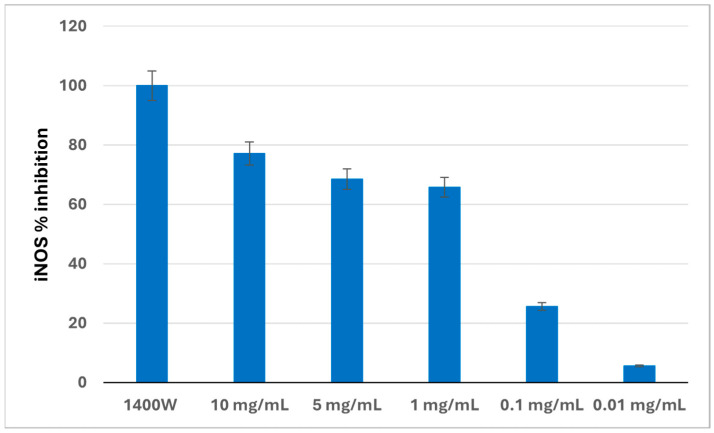
iNOS inhibition by **MP30B**. Results are the mean ± SD of three experiments. The control NOS reactions were performed without an inhibitor (0% inhibition) 1400 W 1 mM was used as the positive control (100% inhibition).

The effects of **MP30B** on the iNOS activity were evaluated by incubating a 0.01–10 mg/mL concentration range of the phenolic complex with the enzyme and then detecting the L-Citr content, which is directly related to the produced nitric oxide (NO) amounts [47]. The potent and selective iNOS inhibitor 1400 W was used as the reference compound (100% inhibition). Figure 5 reports the calculated iNOS percentage inhibition values at each **MP30B** assayed concentration. A dose-dependent enzyme inhibition was obtained, with an inhibition range of 5–77%. A percentage value of 63% was reached at a concentration of 1 mg/mL, and the iNOS IC_50_ was 0.18 ± 0.05 mg/mL.

### 3.5. Antimicrobial Activity

**MP30B** activity was examined according to the Clinical Laboratory Standards Institute (CLSI) recommendations [36], using the broth microdilution method against different streptococcal strains associated with IE. The results are expressed as the minimum inhibitory concentration (MIC) and minimum bactericidal concentration (MBC) for **MP30B** and the reference antibacterial levofloxacin and are shown in Table 2. The obtained data corroborated the antistreptococcal activity of **MP30B** against all the strains under investigation. Indeed, the MIC values were in the range of 4.0–16.2 mg/mL. Fascinating is the effect against the strains *Streptococcus pneumoniae* (ATCC 10015) (responsible for the *Streptococcus pneumoniae* IE, a community-acquired disease that mainly affects native aortic valves), and the streptococcus strain from clinical isolation *Streptococcus pyogenes FL* for which a MIC value of 2.0 mg/mL was calculated. 

A significant effect was also obtained against *Streptococcus pyogenes* (ATCC 19615) and *Streptococcus salivarius* (ATCC 13419) (associated with subacute endocarditis), with a MIC amounting to 4.0 mg/mL. A MIC value of 8.1 mg/mL was instead achieved for *Streptococcus mutans* (ATCC 25175) and *Streptococcus sanguinis* (ATCC 10556). Notably, for all strains considered, it is sufficient to double the MIC to obtain a bactericidal effect; indeed, the MBC value was always twice the calculated MIC value.

## 4. Discussion

Cardiovascular diseases represent one of the leading causes of death in Western countries [48]. The risk increases as cardiovascular problems are often associated with other pathologies, such as diabetes and metabolic syndrome, to name the most common ones [49]. It, therefore, becomes of primary importance to implement all possible strategies to prevent and/or slow down the onset of phenomena linked to functional alterations of the cardiovascular system. Correct nutrition is one of the most important prevention approaches [50]. In this context, the Mediterranean diet is recognized worldwide as a “source of well-being” [51,52]. Among all the foods that are part of the Mediterranean diet, extra virgin olive oil (EVOO) plays a key role due to its nutritional properties, mainly given by the lipid component, but also due to the small concentration of hydrophilic secondary metabolites that are distributed in the EVOO during the extraction process to which a vital health action is attributed [52,53,54,55,56,57]. This hydrophilic fraction, small and contained in EVOO and mainly made up of phenolic structures, is highly concentrated in olive mill wastewater and represents waste from the food chain that needs to be disposed of. Also, considering the extraction process yields, the waste amount is not irrelevant [58]. With a view to the transition from a linear to a circular economy in the agri-food chain and to valorize by-products [59], our research group has been working consistently on extracts obtained from waste from the agri-food chain [10] also for cardiovascular applications [19]).

MOMASTs are commercial products obtained from the OMWW for the production of EVOO from *Olea europaea* L. olives of the *Coratina* variety. **MP30B** represents a fraction of MOMAST^®^, a patented natural phenolic complex [23] enriched in HT (more than 40 mg/g of extract).

The polyphenols contained in EVOO are considered powerful antioxidants. In particular, HT is described as a “cardioprotective molecule” due to its well-known antioxidant properties [41]. These healthy properties have also been confirmed by EFSA, which recommends a daily intake of 20 g (two tablespoons) of EVOO containing at least 5 mg of HT and its derivatives (e.g., oleuropein complex and tyrosol) to obtain the claimed effect specified in the EU Regulation 1924/2006 [60]. In light of this evidence and considering that the same polyphenols reported in the cited claim are present in high amounts in the olive mill wastewater, we studied **MP30B** on some targets linked to cardiac disorders, such as the effects on cardiovascular parameters, the scavenger action, the anti-inflammatory effect and the action on iNOS. Moreover, we also considered the antimicrobial action of the most common microorganisms (streptococcus) responsible for cardiac infections.

The scavenging properties of the polyphenols contained in the various fractions of MOMAST were already known in the literature [9,22,61,62,63]. **MP30B** contains over 40% of HT and other important bioactive phenols. In addition, it is worth noting that the amount of tyrosol present (about 9 mg/g) is probably metabolized in vivo into HT by two isoforms of P450, increasing the antioxidant effect [64]. In addition, “in vivo” studies on rats demonstrate that HT and its metabolites accumulate in a dose-dependent manner in many tissues, including the liver and kidney [65]. In addition, the antioxidant properties of HT favor the reduction of LDL oxidation [66] and promote decreased fat accumulation in the liver [67], contributing to improving cardiovascular risk.

Moreover, cardiovascular effects are reported mainly due to oleuropein and HT in olive oil and by-products [42]. These properties have also been described for a leaf extract and attributed to the presence of oleuropein (215.1 ± 1.64 mg/g of extract) [19]. Oleuropein exerts its cardiovascular properties mainly through the modulation of L-type calcium channels [68]. In particular, the leaf extract shows a significant intrinsic negative inotropic activity with a potency of 0.14 mg/mL and a negative chronotropic action of around 40% at 1mg/mL. The extract, rich in oleuropein, has a vasorelaxing action with IC_50_ = 5.15 mg/mL. On the contrary, and in line with previous results, **MP30B**, which contains a small amount of oleuropein, has a reduced action on all parameters considered: it maintains a negative inotropic effect with similar potency (EC_50_ = 0.21 mg/mL) (a in Table 1), the intrinsic negative chronotropic activity is around 10% at 1 mg/mL, and the vasorelaxing effect is 11% a 10 mg/mL (b in Table 1).

The actions observed are less than those produced by nifedipine, a well-known L-type calcium channel modulator, taken as a positive control. Clinical studies state that supplementation with oleuropein (100 mg) and HT (20 g)/day helps control blood pressure [69]. Our data seem to confirm that the action of **MP30B** is mainly due to the presence of HT. The concentration of oleuropein is low; consequently, the action via L-type calcium channels is reduced (c in Table 1, Figure 1). In addition, the literature seems to agree on the ability of HT to increase intracellular calcium; this could explain the ability of **MP30B** to increase potassium-induced contraction without modifying its potency [70].

Large arteries such as the aorta are elastic and convey blood to the periphery, transforming the rhythmic impulse from the heart into a continuous flow. The increase in aortic stiffness leads to a progressive loss of the ability to relax and contract. If the arteries are stiffer, the heart works harder. The increase in stiffness is physiological with aging, but it is accelerated by external factors mainly linked to lifestyle, smoking, and diet. Therefore, the vessels’ stiffness represents a risk factor, favoring the deposition of materials such as cholesterol, which contribute to accelerating the process.

Most researchers agree that arterial stiffness precedes increased blood pressure [71]. The increase in blood pressure, especially in the elderly, can be mediated by arterial stiffness and is not necessarily related to other pathologies [72]. It, therefore, becomes essential to slow down this process, and diet plays a key role. HT can counteract oxidative stress, vascular aging, and arterial stiffness [73]. Even though the evaluation of the elastance of the different tissues presents an important limitation linked to the impossibility (due to the size of the aorta and the number of guinea pigs available) of verifying the repeatability of the measurement, the curves obtained demonstrate the feasibility of the method and its effectiveness in evaluating the elastic-structural characteristics of the various tissues of interest, to understand whether different pharmaceuticals have effects not only on their spontaneous and induced motility but also on their biomechanical characteristics. From the limited tests, it emerges that for low forces (low pressures), **MP30B** slightly reduces the stiffness of the thoracic aorta, suggesting a possible arterial pressure lowering and a better energetic coupling with a normal (not pathological) ventricle [74]. **MP30B** was studied in comparison to Nifedipine, a well-known antihypertensive drug that acts by blocking the L-type calcium channel [75]. Calcium modulators help slow/reduce arterial stiffness [76]. Nifedipine does not modify the arterial stiffness.

Moreover, the results of spontaneous contractility with **MP30B** highlight an increase in the circular contraction (tone). Arterial circumference decreases, tissues become less stretched, arterial elastance improves, and stiffness reduces. The low number of studied samples limits this evidence, and further deepening is necessary.

HT plays an important role in blood pressure control [77]. The antihypertensive properties of HT are mainly due to its ability to act as a scavenger for reactive oxygen species and to act directly on the vascular endothelium.

Due to multiple factors, endothelial damage is related to and/or precedes the onset of many cardiovascular diseases [78,79]. Many natural compounds present in foods can prevent and/or counteract endothelial dysfunction and, consequently, related diseases [80].

**MP30B** has no cytotoxic effects, given the presence of high concentrations of HT. The pretreatment of HUVEC cells with **MP30B** has protective effects against the action of hydrogen peroxide, confirming the protective and possible preventive action of **MP30B** for cardiovascular disease. Given the known anti-inflammatory effects of HT [81], we studied **MP30B** toward the modulation of TNF-α endothelial tissue through the expression of VCAM-1, a pro-inflammatory protein highly expressed after NF-kB signaling activation (Figure 4), strengthening the protective properties of **MP30B** in the prevention of cardiovascular diseases.

In the human body, the free radical NO is a ubiquitous messenger involved in various physiological functions, pivotal in regulating vascular homeostasis. NO is generated from L-arginine (L-Arg) by three different isoforms of NOS: neuronal (nNOS), inducible (iNOS), and endothelial NOS (eNOS). Although dysfunctional eNOS is considered the main isoform involved in cardiovascular diseases, iNOS also plays an important role because its upregulation contributes to activating the pro-inflammatory cascade. In particular, a dysregulated or high iNOS activity may generate endothelial dysfunction and all pathological states characterized by inflammation, with an unfavorable oxidant versus antioxidant balance through the production of an excess of NO [82]. Thus, inhibiting iNOS could represent an alternative strategy for cardiovascular protection [83]. In recent years, in addition to chemical approaches, several natural polyphenol-based extracts have shown iNOS inhibitory effects, thus preventing the excessive production of NO [84]. Among all polyphenols, HT inhibited the expression of iNOS and COX-2, depending on the concentration [85]. Based on these considerations, we tested the polyphenolic extract **MP30B** for its ability to inhibit the iNOS enzyme through an HPLC method previously reported [35]. The obtained results were encouraging (Figure 5), as an inhibition was observed incubating the iNOS with different concentrations of the extract, ranging from 0.01 to 10 mg/mL. In particular, an IC_50_ 0.18 ± 0.05 mg/mL was determined, in agreement with previous studies on HT, the main component of **MP30B**, in which the inhibition of iNOS-derived NO overproduction induced by bacterial lipopolysaccharide (LPS) has been reported [86]. Our results suggest that **MP30B** can also interfere with inflammatory processes affecting the cardiovascular system by counteracting the deleterious effects caused by uncontrolled NO biosynthesis.

Last but not least, infective endocarditis (IE) should not be underestimated. IE is a critical medical condition with an in-hospital mortality of around 20%, varying across different microbiological etiologies [87]. Even though it occurs rarely, it has a high morbidity, which is associated with high mortality. The aging of the population and the consequent increase in the use of implantable cardiac devices, the most frequent of which are heart valves, have significantly changed the epidemiology of this pathology [88]. Streptococci are among the most frequent causes of IE [89], and some streptococcal species have been reported to be more associated with IE than others [90]. Since streptococci are a heterogeneous group of bacteria with more than 50 species [91], the mortality in IE patients due to different streptococci might vary substantially between species. The treatment for streptococcal infectious endocarditis involves the prolonged intravenous infusion of β-lactams at a high dosage [92]. However, current concerns on the emergence of antimicrobial resistance driven by the excess use of broad-spectrum antibiotics, as well as the risk of *Clostridium difficile* infections during prolonged treatment with third-generation cephalosporins, advocate against the use of these β-lactam agents for the treatment of penicillin-susceptible streptococci, except under rare circumstances [92]. Indeed, in recent years, due to the increasing resistance of pathogenic microorganisms to chemical antimicrobials and the side effects of these compounds, research on natural products has been considered to discover new antibacterial sources. Several studies report the activity of compounds of natural origin with high polyphenol content against streptococci [93,94,95]. This work explores the potential activity of **MP30B** against certain strains of streptococci responsible for IE.

Interestingly, **MP30B** has actions on all types of streptococci tested. In particular, it acts on *Streptococcus pyogenes* and, to a greater extent, on *Streptococcus pyogenes* FL obtained from clinical lysates where the probability of resistance is undoubtedly higher. Comparing the results of **MP30B** with those obtained using the reference antibiotic levofloxacin on *Streptococcus pyogenes* ATCC or FL, it is observed that **MP30B** is more potent both as a bactericidal and as a bacteriostatic on Streptococci FL; contrary to what was observed for levofloxacin whose actions are more significant in ATCC bacteria. In addition, streptococci, particularly pyogenes, produce streptolysin, a toxin that binds to white and red blood cells and is toxic to cells and tissues. Studies on polyphenols obtained from *O. europaea*, particularly HT, inhibit this toxin’s hemolytic action [96], further strengthening the protective and/or preventive action of **MP30B**.

## 5. Conclusions

In conclusion, **MP30B**, obtained from Olive Mill Wastewater using a patented technique that allows enrichment in HT, showed a potential application as a food supplement to prevent cardiovascular diseases. The effects detected affect some network nodes that target the main cardiovascular pathologies, especially those related to aging and metabolic syndrome. The weak cardiovascular effects support the possibility of using **MP30B** to prevent cardiovascular disorders as they do not significantly modify cardiac and vascular parameters. At the same time, it exerts a strong action as a scavenger of reactive oxygen species, has an anti-inflammatory effect that combines a reduction in the concentration of iNOS, and contributes to the prevention of heart infections. Studies on the cardiotoxic effects of anti-tumor therapies demonstrate the protective action of HT in breast cancer cardiotoxicity [97], increasing the potential applications of **MP30B**.

## Figures and Tables

**Table 1:** “In vitro” effects of MP30B in guinea pig tissue preparations.

**(a) Cardiac Activity on Guinea Pig Heart Preparations**									
	**Left atrium**			**Right atrium**					
	**inotropy**			**inotropy**			**chronotropy**		
	**IA *^a^*** **(M ± SEM)**	**EC_50_ *^b^***	**95%** **conf lim**	**IA *^a^*** **(M ± SEM)**	**EC_50_ *^b^***	**95%** **conf lim**	**IA *^c^*** **(M ± SEM)**	**EC_50_ *^b^***	**95%** **conf lim**
**MP30B**	75 ± 0.3 (1 mg/mL)	0.21 (mg/mL)	0.16−0.42 (mg/mL)	69 ± 1.3 (1 mg/mL)	0.44 (mg/mL)	0.21−0.56 (mg/mL)	10 ± 0.2 (1 mg/mL)		
**NIF**	97 ± 2.0 (10 μM) (3.5 µg/mL)	0.26 (μM) (0.09 µg/mL)	0.19−0.36 (μM) (0.066−0.12 µg/mL)				85 ± 4.2 (0.1 μM) (0.035 µg/mL)	0.039 (μM) (0.013 µg/mL)	0.031−0.051 (μM) (0.011−0.018 µg/mL)
**(b) Spasmolytic activity on K^+^-depolarized Guinea pig aorta**									
				**IA *^d^*** **(M ± SEM)**	**IC_50_ *^c^***	**95%** **conf lim**			
**MP30B**				11 ± 0.4 (10 mg/mL)					
**NIF**				97 ± 1.4 (1 µM) (0. 35 µg/mL)	0.0064 (µM) (0.0022 µg/mL)	0.0016−0.0093 (µM) (0.0055−0.0032 µg/mL)			
**(c) Antispastic activity on K^+^-induced contraction Guinea pig aorta**									
				**IA *^e^*** **(M ± SEM)**	**EC_50_ *^c^***	**95%** **conf lim**			
**KCl**				70 ± 10	38.42	27.12−42.26			
**KCl + MP30B** (1 mg/mL)				135 ± 12	36.37	25.08−45.38			
KCl				335± 15	32.24	20.05−38.89			
**KCl + NIF** (0.005 µM) (0.0017 µg/mL)				199 ± 11	38.07	26.59−43.45			

*^a^* **IA** (Intrinsic Activity) expressed as a percent decrease in developed tension on the isolated guinea pig’s left atrium driven at 1 Hz and on the guinea pig’s spontaneously beating isolated right atrium, expressed as percent changes from the control (n = 5–6). The concentration indicated in brackets gave the maximum effect. *^b^* Calculated from concentration–response curves to **MP30B** or log concentration–response curves to **NIF** (nifedipine) and **KCl** (Probit analysis by Litchfield and Wilcoxon [38] with n = 6–7). When the maximum effect was <50%, the EC_50_ ino., EC_50_ chrono., and IC_50_ vasorelax. values were not calculated. *^c^* Decrease in atrial rate on Guinea pig spontaneously beating isolated right atrium, expressed as percent changes from the control (n = 7–8). Pretreatment heart rate ranged from 165 to 190 beats/min. The concentration indicated in brackets gave the maximum effect. *^d^* **IA** (Intrinsic Activity) expressed as percent inhibition of calcium-induced contraction on K^+^-depolarized (80 mM) guinea pig aorta smooth muscle. The concentration indicated in brackets gives the maximum effect. *^e^* **IA** (Intrinsic Activity) expressed as mg of contraction induced by K^+^ on guinea pig aorta smooth muscle alone or in the presence of **MP30B** (1 mg/mL) or **NIF** (nifedipine 0.005 µM.

**Table 2:** Minimum Inhibitory Concentration (MIC) and minimum bactericidal concentration (MBC) for **MP30B** and Levofloxacin.

Strain	MP30B (mg/mL)		Levofloxacin (µg/mL)	
	MIC	MBC	MIC	MBC
*Streptococcus mutans* ATCC 25175	8.1	16.2	4	8
*Streptococcus pneumoniae* ATCC 10015	2.0	4.0	2	4
*Streptococcus pyogenes* ATCC 19615	4.0	8.1	0.5	1
*Streptococcus pyogenes* FL	2.0	4.0	1	2
*Streptococcus salivarius* ATCC 13419	4.0	8.1	2	4
*Streptococcus sanguinis* ATCC 10556	8.1	16.2	4	8

## Data Availability

Data available upon request to the corresponding author.

## Supplementary Materials

The following supporting information can be downloaded at: https://www.mdpi.com/article/10.3390/nu16172986/s1, Table S1. Chemical analysis of organic and inorganic residues in the extract **MP30B**; Figure S1. HPLC-VWD chromatogram recorded at 280 nm of the main phenolic compounds present in **MP30B**.
