# Supplementary material for: From Waste to Health: Olive Mill Wastewater for Cardiovascular Disease Prevention
Source: Nutrients. 2024 Sep 4;16(17):2986. doi: 10.3390/nu16172986 (PMC11397552; doi:10.3390/nu16172986)
Supplement: Supplementary file 1 [file nutrients-16-02986-s001.zip › nutrients-3149288-supplementary.pdf]

Table S1. Chemical analysis of organic and inorganic residues in the extract **MP30B**.

Compound	Content (mg/L)	LOD (mg/L)
Inorganic contaminant		
Ammonium	108 ± 25	0.1
Nitric nitrogen	< 0.1	0.1
Nitrous nitrogen	< 0.01	0.01
Total nitrogen	120 ± 30	0.1
Cloride	21.0 ± 1.3	0.5
Fluoride	< 0.05	0.05
Total phosphorus	< 0.01	0.01
Total cyanide	< 0.05	0.05
Aluminum (Al)	< 0.1	0.1
Antimony (Sb)	< 0.005	0.005
Silver (Ag)	< 1	1
Arsenic (As)	< 0.002	0.002
Barium (Ba)	< 0.1	0.1
Boron (B)	< 0.1	0.1
Beryllium (Be)	< 0.01	0.01
Cadmium (Cd)	< 0.002	0.002
Cobalt (Co)	< 0.001	0.001
Total chromium (Cr)	< 0.1	0.1
Hexavalent chromium (Cr VI)	< 0.01	0.01
Iron (Fe)	< 0.1	0.1
Manganese (Mn)	0.10 ± 0.03	0.02
Mercury (Hg)	< 0.0001	0.0001
Molybdenum (Mo)	< 0.1	0.1
Nichel (Ni)	< 0.01	0.01
Lead (Pb)	< 0.01	0.01
Copper (Cu)	0.012 ± 0.003	0.005
Selenium (Se)	< 0.0001	0.0001
Tin (Sn)	< 0.1	0.1
Thallium (Tl)	< 0.001	0.001
Tellurium (Te)	< 0.01	0.01
Vanadium (V)	< 0.01	0.01
Zinc (Zn)	< 0.01	0.01
Calcium (Ca)	18.0 ± 0.9	0.1
Magnesium (Mg)	10.0 ± 0.5	0.1
Potassium (K)	1.40 ± 0.14	0.1
Sodium (Na)	14.0 ± 1.1	0.1
Copper oxide (II)	< 0.1	0.1
Organic contaminant		
Benzene	< 0.002	0.002
Ethyl benzene	< 0.002	0.002
Toluene	< 0.002	0.002
Xylenes	< 0.002	0.002

Styrene	< 0.002	0.002
1,3-Butadiene	< 0.002	0.002
Aromatic polycyclic hydrocarbons		
Acenaphthylene	< 0.001	0.001
Naphthalene	< 0.001	0.001
Acenaphthene	< 0.001	0.001
Anthracene	< 0.001	0.001
Benzo[a]anthracene	< 0.001	0.001
Fluorene	< 0.001	0.001
Phenanthrene	< 0.001	0.001
Fluoranthrene	< 0.001	0.001
Pyrene	< 0.001	0.001
Chrysene	< 0.001	0.001
Benzo[e]fluoranthrene	< 0.001	0.001
Benzo[k]fluoranthrene	< 0.001	0.001
Benzo[j]fluoranthrene	< 0.001	0.001
Benzo[e]pyrene	< 0.001	0.001
Benzo[a]pyrene	< 0.001	0.001
Benzo[g,h,i]perylene	< 0.001	0.001
Dibenzo[a,h]anthracene	< 0.001	0.001
Indeno[1,2,3-cd]pyrene	< 0.001	0.001
Perylene	< 0.001	0.001
Dibenzo[a,h]pyrene	< 0.001	0.001
Dibenzo[a,l]pyrene	< 0.001	0.001
Dibenzo[a,e]pyrene	< 0.001	0.001
Hydrocarbons		
Total hydrocarbons	1.05 ± 0.11	0.1
Volatile organic compounds (VOC)		
Trichlorofluoromethane	< 0.001	0.001
Methylene chloride	< 0.001	0.001
Trans-1,2-dichloroethene	< 0.001	0.001
1,1-dichloroethane	< 0.001	0.001
2,2-dichloropropane	< 0.001	0.001
Bromochloromethane	< 0.001	0.001
Trichloromethane	< 0.001	0.001
1,1,1-trichloroethane	< 0.001	0.001
Carbon tetrachloride	< 0.001	0.001
1,1-dichloropentene	< 0.001	0.001
1,2-dichloroethane	< 0.001	0.001
Trichloroethylene	< 0.001	0.001
1,2-dichloropropane	< 0.001	0.001
Dichloromethane	< 0.001	0.001
Bromodichloromethane	< 0.001	0.001
Cis-1,3-dichloropropene	< 0.001	0.001
Trans-1,3-dichloropropene	< 0.001	0.001
Tetrachloroethylene	< 0.001	0.001
1,3-dichloropropane	< 0.001	0.001
Bibromochloromethane	< 0.001	0.001

1,2-dibromoethane	< 0.001	0.001
Chlorobenzene	< 0.001	0.001
Bromoform	< 0.001	0.001
Isopropylbenzene	< 0.001	0.001
Bromobenzene	< 0.001	0.001
1,1,2,2-tetrachloroethane	< 0.001	0.001
1,2,3-trichloropropane	< 0.001	0.001
Propylbenzene	< 0.001	0.001
4-chlorotoluene	< 0.001	0.001
2-clhlorotoluene	< 0.001	0.001
1,2,4-trimethylbenzene	< 0.001	0.001
Terbutylbenzene	< 0.001	0.001
1,3,5-trimethylbenzene	< 0.001	0.001
Sec-butylbenzene	< 0.001	0.001
1,4-dichlorobenzene	< 0.001	0.001
4-isopropyltoluene	< 0.001	0.001
1,3-dichlorobenzene	< 0.001	0.001
1,2-dichlorobenzene	< 0.001	0.001
Butylbenzene	< 0.001	0.001
1,2-dibromo-3-chloropropane	< 0.001	0.001
1,2,4-trichlorobenzene	< 0.001	0.001
Chloromethane	< 0.001	0.001
Dichloromethane	< 0.001	0.001
Vinyl chloride	< 0.001	0.001
1,1-dichloroethylene	< 0.001	0.001
1,2-dichloroethylene	< 0.001	0.001
1,1,2-trichloroethane	< 0.001	0.001
Tribromomethane	< 0.001	0.001
p-Phtalic acid	< 0.01	0.01
2-chlorophenol	< 0.001	0.001
2,4-dichlorophenol	< 0.001	0.001
2,4,6-trichlorophenol	< 0.0001	0.0001
Persistent organic pollutants (POPS)		
Aldrin	< 0.001	0.001
α-HCH	< 0.001	0.001
β-HCH	< 0.001	0.001
Chlordane	< 0.001	0.001
Dieldrin	< 0.001	0.001
Alaclor	< 0.001	0.001
Atrazine	< 0.001	0.001
Hexachlorobenzene	< 0.001	0.001
Heptabromodiphenylbenzene	< 0.001	0.001
Hexachlorohexane	< 0.001	0.001
Hexabromodiphenylether	< 0.001	0.001
Lindane	< 0.001	0.001
Mirex	< 0.001	0.001
Pentabromodiphenylether	< 0.001	0.001
Pentachlorobenzene	< 0.001	0.001

Tetrabromodiphenylether	< 0.001	0.001
Decabromodiphenylether	< 0.001	0.001
Toxaphene	< 0.001	0.001
DDD, DDT, DDE	< 0.01	0.01
PCB, PCT	< 0.5	0.5

Analyses were carried out by an accreditated laboratory (ACCREDIA LAB n. 0648) following UNI CEI ISO/IEC 17025:2018 regulations.

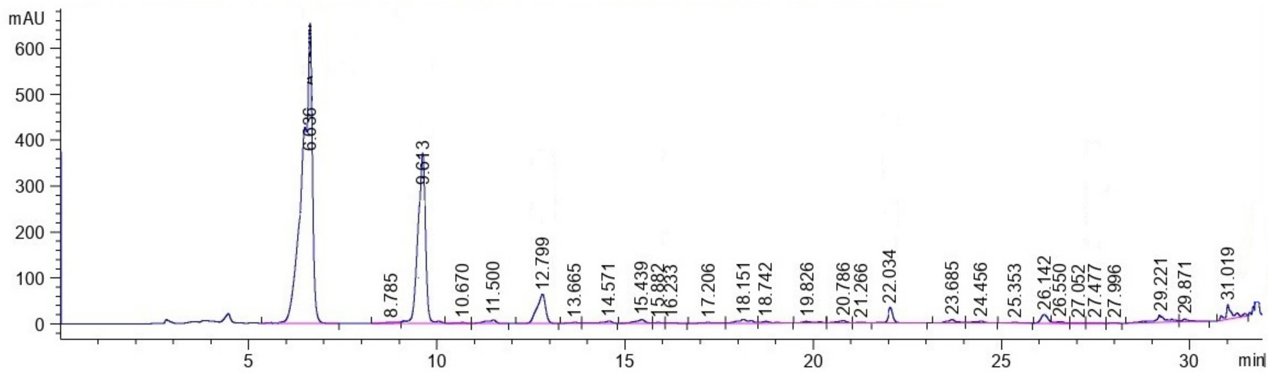

Figure S1. HPLC-VWD chromatogram recorded at 280 nm of the main phenolic compounds present in **MP30B**. Gallic acid (6.636 min), 3-hydroxytyrosol (9.613 min), tyrosol (12.799 min), verbascoside (22.034 min), oleuropein (27.477 min).
